# Identification of Conserved B-Cell Epitope Candidates Associated with Neutralizing Activity of Bovine Coronavirus Spike Protein

**DOI:** 10.3390/ani16162598

**Published:** 2026-08-20

**Authors:** Yunxin Ren, Hua Yue, Cheng Tang, Xi Chen

**Affiliations:** Key Laboratory of Animal Medicine of Sichuan Education Department, Southwest Minzu University, Chengdu 610041, China; 17614855357@163.com (Y.R.); 22100037@swun.edu.cn (H.Y.); tangcheng101@163.com (C.T.)

**Keywords:** bovine coronavirus, spike protein, B-cell epitope candidates, ferritin nanoparticle, neutralizing antibody

## Abstract

Bovine coronavirus (BCoV) is a major cause of respiratory and enteric diseases in cattle, resulting in substantial economic losses to the global cattle industry. Next-generation epitope-focused vaccines require conserved neutralizing determinants to achieve broad protective efficacy across circulating variants. Here, we applied a reverse-vaccinology and immunoinformatics-guided strategy to identify conserved linear B-cell epitope candidates on the BCoV spike protein. Using ABCPred (16 aa; threshold score 0.51), 139 putative linear B-cell epitopes were predicted, and 10 candidates (B1–B10) were selected based on antigenicity, allergenicity/toxicity filters, and Shannon-entropy-based conservation (≥95%). Each candidate was displayed on *Helicobacter pylori* ferritin and TEM confirmed the formation of self-assembled nanocages. BALB/c mice (n = 6 per group) were subcutaneously immunized with 50 μg of each epitope–ferritin fusion protein emulsified 1:1 with Montanide ISA 201 in a prime–boost regimen (at a 14-day interval), and sera were collected 14 days after the booster immunization. All 10 constructs induced BCoV-specific binding IgG (endpoint titers ranging from 1:2667 to 1:11,733), but only B1, B2, B4, B6, B7, and B8 elicited detectable in vitro neutralizing activity against both representative strains, whereas B3, B5, B9, and B10 remained below the detection limit (<1:8). Neutralizing titers were 1:24–1:99 for the enteric strain XHD4 and 1:27–1:88 for the respiratory strain HXD1, with no significant difference observed between the two strains (*p* > 0.05). Overall, these findings identify six conserved B-cell epitope candidates capable of eliciting neutralizing antibody responses against both representative enteric and respiratory field strains, providing experimental evidence to support epitope-focused BCoV vaccine design.

## Simple Summary

Bovine coronavirus (BCoV) causes substantial economic losses to the global cattle industry through respiratory and enteric infections. Current vaccines confer limited cross-protection against antigenically divergent BCoV strains. In this study, we used immunoinformatics to predict 10 conserved linear B-cell epitope candidates (B1 to B10) from the BCoV spike protein and displayed each on self-assembling ferritin nanoparticles. All 10 constructs induced BCoV-specific IgG antibodies in mice, but only six (B1, B2, B4, B6, B7, and B8) elicited detectable neutralizing antibodies against both an enteric strain (XHD4) and a respiratory strain (HXD1), with comparable titers observed between the two strains (*p* > 0.05). These results identify six conserved epitope candidates capable of eliciting neutralizing antibody responses against both representative enteric and respiratory field strains, providing a solid foundation for epitope-focused BCoV vaccine design.

## 1. Introduction

Bovine coronavirus (BCoV) is a major pathogen associated with neonatal calf diarrhea, winter dysentery in adult cattle, and the bovine respiratory disease complex, inflicting substantial economic damage on the global livestock industry [1,2]. Like other coronaviruses, BCoV undergoes continuous genetic and antigenic variation, particularly in the spike (S) glycoprotein, which serves as the major target of neutralizing antibodies [3,4,5]. Consequently, vaccine-induced humoral immunity may not always provide consistent protection against antigenically divergent strains. This protective efficacy remains highly variable, being influenced by host age, tissue tropism (respiratory versus enteric tract), and antigenic mismatch between vaccine strains and circulating wild-type lineages [6,7]. Therefore, the identification of conserved antigenic regions capable of eliciting broad neutralizing antibody responses across different clinical strains is important for the rational design of next-generation BCoV vaccines.

Epitope-focused immunogen design has emerged as a promising strategy to direct immune responses toward functionally relevant and relatively conserved viral regions. Compared with whole-antigen or whole-virus formulations, this approach can diminish non-protective or decoy antibody responses against hypervariable regions while focusing humoral immunity onto epitopes associated with viral neutralization [8,9]. As neutralizing antibodies are a key correlate of coronavirus protection, defining functional neutralizing epitopes is central to epitope-based vaccine design [10,11]. Evidence from SARS-CoV-2 studies indicates that conserved spike-epitope immunogens can induce broadly neutralizing humoral immunity, offering protection against continually emerging variants [12]. Structural and antigenic analyses of HCoV-OC43 further show that neutralizing antibodies recognize defined antigenic sites on the OC43 spike, including regions that display varying degrees of conservation across OC43 isolates, thereby supporting the feasibility of designing epitope-focused immunogens for broad-spectrum protection against viral diversification [11]. Notably, HCoV-OC43 is genetically closely related to BCoV and is believed to have originated from cattle. Therefore, HCoV-OC43 provides a valuable reference for applying epitope-targeting vaccine strategies to BCoV [13].

The BCoV S protein mediates host cell entry and represents the dominant target of neutralizing antibodies [14], placing it at the center of epitope-based vaccine design [15,16]. Although the S protein contains major conformational neutralizing domains, these sites are highly dependent on native conformation and susceptible to sequence variations [17]. This structural and antigenic plasticity complicates the use of classical conformational regions as broadly protective vaccine targets. Conserved neutralizing epitopes, by contrast, may offer more durable and mutation-tolerant targets, but their distribution and functional relevance in the BCoV S protein remain poorly characterized. In the present study, we aimed to screen conserved B-cell epitope candidates from the BCoV S protein and evaluate their immunogenicity and neutralizing potential using a ferritin nanoparticle display platform.

## 2. Materials and Methods

### 2.1. B-Cell Epitope Prediction

To identify potential B-cell epitopes within the BCoV S protein, we employed immunoinformatics approaches for sequence prediction. Linear B-cell epitopes were selected for peptide vaccine design because they can be recognized independently of native tertiary conformation, retaining reactivity even after protein denaturation. We focused on the S protein sequence of the BCoV/SWUN/XHD4/2022 strain (GenBank accession: OR621178), a prevalent Chinese isolate, to identify neutralizing epitopes relevant to circulating strains. We utilized the ABCPred online prediction tool (https://webs.iiitd.edu.in/raghava/abcpred/, accessed on 15 October 2024) for linear B-cell epitope prediction from the S protein sequence. ABCPred, a widely recognized tool employing a recurrent neural network model, predicts potential epitope regions by learning features from known B-cell epitope datasets, offering robust predictive accuracy. Peptide length was set to 16 amino acids (aa) and a threshold score of 0.51 was applied, thereby selecting peptides with a probability greater than 0.51 of being a B-cell epitope. This threshold effectively balanced prediction sensitivity and specificity, ensuring high reliability of selected candidates [18]. All high-scoring candidate epitopes were subsequently advanced to downstream screening and immunogenicity evaluation.

### 2.2. B-Cell Epitope Screening

The resulting peptides were assessed for antigenicity, allergenicity, and toxicity using VaxiJen (https://www.ddg-pharmfac.net/vaxijen/VaxiJen/VaxiJen.html, accessed on 16 October 2024), AllerTOP (https://www.ddg-pharmfac.net/allertop_test/, accessed on 16 October 2024), and ToxinPred (https://webs.iiitd.edu.in/raghava/toxinpred/, accessed on 16 October 2024), respectively. Peptides predicted to be antigenic (VaxiJen score ≥ 1.0), non-allergenic, and non-toxic were retained. Conservation among BCoV strains was evaluated using available BCoV *S* gene sequences retrieved from GenBank. Sequences were filtered to retain complete or nearly complete S coding sequences annotated as BCoV in GenBank. Sequences containing extensive ambiguous residues, premature stop codons, or abnormal lengths were excluded. Redundant and low-quality sequences were removed before downstream analysis. After quality filtering, 492 BCoV S sequences were retained for conservation analysis, including 475 sequences from bovine hosts and 17 sequences from non-bovine or uncertain hosts.

The nucleotide sequences were translated into aa sequences and aligned using MAFFT v7.0 (https://www.ebi.ac.uk/Tools/msa/mafft/, accessed on 20 October 2024) with default parameters. The alignment was manually inspected to remove sequences with apparent alignment errors. Aa variability at each position of the S protein was quantified using Shannon entropy according to the following formula:

$$H\left(x\right)=-\sum_{i=1}^{N}p_{i}\log_{2}p_{i}$$

where H(x) denotes the Shannon entropy at amino acid position x, and pi represents the frequency of the i-th amino acid type, including standard amino acids and gaps. A value of H = 0 indicates complete conservation. When pi = 0, the term pi log_2_pi was set to 0. The mean entropy of each epitope was calculated by averaging the entropy values of all residues within the epitope [19]. Residue-level conservation was defined as the frequency of the dominant amino acid at each position, and epitope-level conservation was defined as the average residue-level conservation across all residues within the epitope. Epitopes were considered broadly conserved and selected if their epitope-level conservation (average dominant aa frequency) was ≥95%.

### 2.3. Design and Construction of B-Cell Epitope Display on Ferritin Protein

Selected candidate linear B-cell epitopes were genetically fused as a single copy (without tandem repeats) to the N-terminus of *Helicobacter pylori* ferritin subunits (GenBank accession: WP_000949190.1) via a flexible (GGGS)_2_ linker to enhance spatial flexibility and epitope exposure. The ferritin scaffold contained the previously reported N19Q substitution [20]. Structural models of the recombinant fusion proteins were generated using AlphaFold 3 (https://alphafoldserver.com/, accessed on 22 October 2024) and visualized in PyMOL v2.5 to assess the surface exposure of the inserted epitopes. The designed fusion genes were codon-optimized for *Escherichia coli* expression, synthesized, and cloned into the *pET-28a* vector by Tsingke Biotechnology Co., Ltd. (Chengdu, China). The recombinant plasmids were subsequently transformed into *Escherichia coli* BL21(DE3) for protein expression.

### 2.4. Expression and Purification of Fusion Proteins

The *pET-28a* plasmids encoding epitope-ferritin fusion proteins were transformed into *Escherichia coli* BL21(DE3). Positive transformants were cultured in Luria–Bertani (LB) medium containing 50 µg/mL kanamycin at 37 °C with shaking at 200 rpm until the OD_600_ reached approximately 0.6. The cultures were then shifted to 16 °C, and protein expression was induced with 0.1 mM isopropyl-β-D-1-thiogalactopyranoside (IPTG) for 16 h. Cells were harvested by centrifugation at 4000× *g* for 15 min at 4 °C and resuspended in ice-cold phosphate-buffered saline (PBS, pH 7.4), at 5 mL per gram of wet cell pellet. The suspension was sonicated on ice (400 W, 3 s pulse, 6 s rest), until it became translucent, followed by centrifugation at 12,000× *g* for 15 min at 4 °C to remove cell debris. The soluble fraction was collected and subjected to Ni-NTA affinity purification using Ni NTA Beads 6FF (Smart-Lifesciences Biotech, Changzhou, China). Eluted fractions were dialyzed against pre-chilled PBS at 4 °C using dialysis tubing with a 10 kDa molecular weight cut-off. The dialysis buffer was replaced every 4 h, with at least two buffer changes over a minimum of 12 h. Protein concentrations were determined the Bicinchoninic Acid Protein Assay Kit (Thermo Scientific, Waltham, MA, USA). Protein samples collected during expression and purification were analyzed by SDS-PAGE. Samples were mixed with 5× loading buffer, boiled at 95 °C for 5 min, separated on a 12% polyacrylamide gel, and visualized by Coomassie Brilliant Blue R-250 staining. Endotoxin was removed using an endotoxin removal kit (Sigma-Aldrich, St. Louis, MO, USA).

### 2.5. Western Blot Analysis

For Western blot analysis, proteins separated by SDS-PAGE were transferred onto a polyvinylidene fluoride (PVDF) membrane. The membrane was blocked with 5% non-fat milk in Tris-buffered saline containing Tween 20 (TBST) for 1 h at 37 °C. Subsequently, the membrane was incubated overnight at 4 °C with a mouse anti-His tag monoclonal antibody (1:5000, Sanying, Wuhan, China). After being washed with TBST, the membrane was incubated with a horseradish peroxidase (HRP)-conjugated goat anti-mouse IgG secondary antibody (1:5000, BOSTER, Wuhan, China) for 1 h at room temperature. Finally, protein bands were detected using an enhanced chemiluminescence substrate (Epizyme, Shanghai, China) and imaged using a Bio-Rad ChemiDoc MP Imaging System (Bio-Rad, Hercules, CA, USA).

### 2.6. Transmission Electron Microscopy (TEM) of Fusion Proteins

The self-assembly of BCoV epitope-ferritin fusion proteins into uniform nanoparticles was verified by TEM using 2% phosphotungstic acid negative staining. Purified and dialyzed protein samples (0.3 mg/mL) were adsorbed onto carbon-coated copper grids, negatively stained, and visualized under a transmission electron microscope (JEM-1400FLASH, JEOL, Tokyo, Japan). Particle diameters were measured from representative TEM images using ImageJ software (v1.6.0). Briefly, the scale was calibrated based on the scale bar in each image, and individual nanoparticles with clearly defined boundaries were manually selected for diameter measurement. At least 50 particles were analyzed for each sample, and the particle size distribution was presented as the mean ± standard deviation (SD).

### 2.7. Immunization of Mice

Female BALB/c mice (6 weeks old, n = 6 per group) were purchased from Dossy Experimental Animals Co., Ltd. (Chengdu, China), randomly allocated into groups, and immunized subcutaneously using a prime-boost regimen at a 14-day interval. The sample size of six mice per group was selected in accordance with established precedents in ferritin-based vaccine studies and ethical principles regarding the reduction of animal use [21,22]. Mice were administered 50 μg of the corresponding epitope–ferritin fusion protein or an empty ferritin scaffold as a carrier control. Each antigen was emulsified 1:1 (*v*/*v*) with Montanide ISA 201 adjuvant (Seppic, Paris, France). A PBS-adjuvant group was included as the vehicle control. Sera were collected 14 days after boosting and stored at −80 °C for further analysis. All animal experiments were approved by the Institutional Animal Care and Use Committee of Southwest Minzu University, and all animals were handled in accordance with the approved protocol (Approval Code SMU2024010961; approval date 6 June 2024).

### 2.8. Indirect ELISA and Neutralization Assay

After immunization, serum BCoV-specific IgG antibody levels were measured by indirect ELISA. Briefly, 96-well plates (NEST, Wuxi, China) were coated overnight at 4 °C with ultracentrifuged, β-propiolactone-inactivated whole BCoV (XHD4 strain, GenBank accession: OR621178, 10^8^ TCID_50_/mL). After blocking with 2% BSA at 37 °C for 1 h, two-fold serially diluted sera (starting at 1:100) were added and incubated at 37 °C for 1 h. The plates were then incubated with HRP-conjugated goat anti-mouse IgG (1:5000, BOSTER, Wuhan, China) at 37 °C for 1 h. Finally, TMB substrate was added and incubated at 37 °C for 10 min, the reaction was stopped with 2 M H_2_SO_4_, and absorbance was measured at 450 nm. The specific antibody titer was defined as the highest serum dilution yielding a P/N ratio ≥2.1 (P: sample OD_450_ nm, N: mean OD_450_ nm of negative controls).

For the micro-neutralization assay, collected sera were heat-inactivated at 56 °C for 30 min and serially diluted twofold in DMEM. This assay served as a functional readout to determine whether epitope-induced antisera could neutralize BCoV strains representing the two principal clinical forms of infection, the enteric and the respiratory forms. Prior to the assay, the enteric XHD4 strain (GenBank accession: OR621178, 10^6^ TCID_50_/mL, isolated from neonatal calf diarrhea) and the respiratory HXD1 strain (GenBank accession: OR612022, 10^5^ TCID_50_/mL, isolated from the bovine respiratory tract) were each treated with 1 µg/mL trypsin at 37 °C for 30 min to activate viral entry, after which the trypsin-treated virus stocks were diluted to 100 TCID_50_ per 50 µL in DMEM. The two strains were selected because they represent the two major clinical manifestations and transmission routes of BCoV infection and display minor sequence divergence across the *S* gene despite belonging to the same genotype; phylogenetic analysis confirmed that both strains fall within genotype GIIb (Supplementary Figure S1), the predominant lineage circulating in China, sharing 98.8% nucleotide identity and 98.5% aa identity in the *S* gene. The diluted sera (50 µL) were mixed with an equal volume of the activated virus suspension (100 TCID_50_) and incubated at 37 °C for 1 h. The serum–virus mixtures were then inoculated onto pre-seeded HCT-8 cell monolayers in 96-well plates, with four replicate wells per serum dilution. Cytopathic effect (CPE) was monitored daily, and neutralizing antibody titers were calculated using the Reed–Muench method, defined as the reciprocal of the highest serum dilution capable of protecting 50% of the cells from viral infection. Neutralizing antibody titers were log_2_-transformed prior to statistical analysis. Samples below the detection limit of 1:8 were assigned a nominal value of 1 for statistical purposes, corresponding to 0 after log_2_ transformation.

### 2.9. Epitope Mapping and Validation on the BCoV S Protein

The 3D homotrimeric structure of the BCoV S protein (GenBank: OR621178) was modeled using AlphaFold 3 and visualized using PyMOL (v2.5). The epitope candidates that elicited neutralizing responses were mapped onto the S homotrimer and highlighted in distinct colors.

To confirm that the neutralizing activity of the harvested antisera was mediated by specific binding to the native BCoV S protein, Western blotting was performed using purified virions. BCoV virions were purified by ultracentrifugation of infected cell culture supernatant as described previously [23]. Briefly, virus-infected cell cultures (XHD4 strain) underwent two freeze–thaw cycles at −80 °C, after which the supernatant was collected and centrifuged at 3000× *g* for 10 min at 4 °C to remove HCT-8 cell debris. The clarified supernatant was layered onto a 35% (*w*/*v*) sucrose cushion prepared in TNC buffer (50 mM Tris, 150 mM NaCl, 10 mM CaCl_2_, 0.02% NaN_3_, pH 7.4) and centrifuged at 112,700× *g* for 2 h at 4 °C. The viral pellet was resuspended in TNC buffer, centrifuged at 107,200× *g* for 2 h at 4 °C, and washed twice with sterile PBS (1×, pH 7.4). Purified virions (25 μg total protein per lane) were resolved by SDS-PAGE, and Western blotting was performed as described in Section 2.5. Briefly, transferred membranes were probed with mouse antisera raised against each neutralizing epitope (B1, B2, B4, B6, B7, and B8) as primary antibodies, while antisera raised against ferritin alone and PBS served as negative controls. Based on the previously reported molecular weight range of BCoV S protein domains [24], the detected bands were evaluated against the predicted sizes of the S1 (~84 kDa) and S2 (~65 kDa) cleavage fragments. Detection of specific bands corresponding to these expected sizes confirmed that the neutralizing antibodies specifically recognized the native BCoV S protein on intact virions.

### 2.10. Statistical Analysis

Statistical analyses and graphing were performed using GraphPad Prism 9 (https://www.graphpad.com, accessed on 3 December 2025) and G*Power 3.1 (https://www.psychologie.hhu.de/arbeitsgruppen/allgemeine-psychologie-und-arbeitspsychologie/gpower, accessed on 15 August 2026). Data are presented as the mean ±SD. The sample size of six mice per group was determined based on established protocols in nanoparticle vaccine immunogenicity studies and was strictly guided by the 3R ethical principle (Reduction) to minimize animal numbers without unnecessary animal use. To confirm the statistical reliability of this cohort size, a Post hoc power analysis was conducted using G*Power 3.1. The analysis confirmed that, given the observed large biological effect sizes (Cohen’s d > 2.0), a sample size of n = 6 per group provided a statistical power (1 − β) greater than 0.85 at a significance level of α = 0.05, demonstrating experimental adequate sensitivity to detect statistically significant differences among groups to support subsequent analyses while adhering to ethical standards. To compare the neutralizing efficacy against different BCoV strains, specifically the enteric XHD4 strain (GenBank accession: OR621178, isolated from neonatal calf diarrhea) and the respiratory HXD1 strain (GenBank accession: OR612022, isolated from the bovine respiratory tract), neutralizing antibody titers were log_2_-transformed before statistical analysis to approximate a normal distribution and to stabilize variance across groups. Prior to parametric analysis, the normality of the log_2_-transformed titer data within each group was assessed using the Shapiro–Wilk test, and homogeneity of variances across groups was evaluated using Levene’s test; the data satisfied both the normality (*p* > 0.05) and homogeneity-of-variance assumptions, permitting the use of parametric two-way analysis of variance (ANOVA). The log_2_-transformed titers were then analyzed using two-way ANOVA, followed by Bonferroni’s multiple-comparison test. Differences were considered statistically significant at *p* < 0.05. Statistical significance levels are indicated as follows: ns (not significant, *p* > 0.05), * *p* < 0.05, ** *p* < 0.01, and *** *p* < 0.001.

## 3. Results

### 3.1. Linear B-Cell Epitope Prediction and Screening

Linear B-cell epitopes within the BCoV S protein were predicted using ABCPred, which identified 139 putative linear epitopes (Supplementary Table S1). The predicted sequences were subsequently screened based on their antigenicity, toxicity, allergenicity, and sequence conservation. Based on these criteria, 10 candidate epitopes (B1–B10) were selected for further investigation (Table 1), exhibiting antigenicity scores ranging from 1.035 to 1.491 and conservation levels between 98.37–99.93%. Furthermore, all selected epitopes were predicted to be both non-toxic and non-allergenic.

### 3.2. Structural Design, Construction, and Expression of the Epitope-Ferritin Nanoparticles

**Figure 1:**
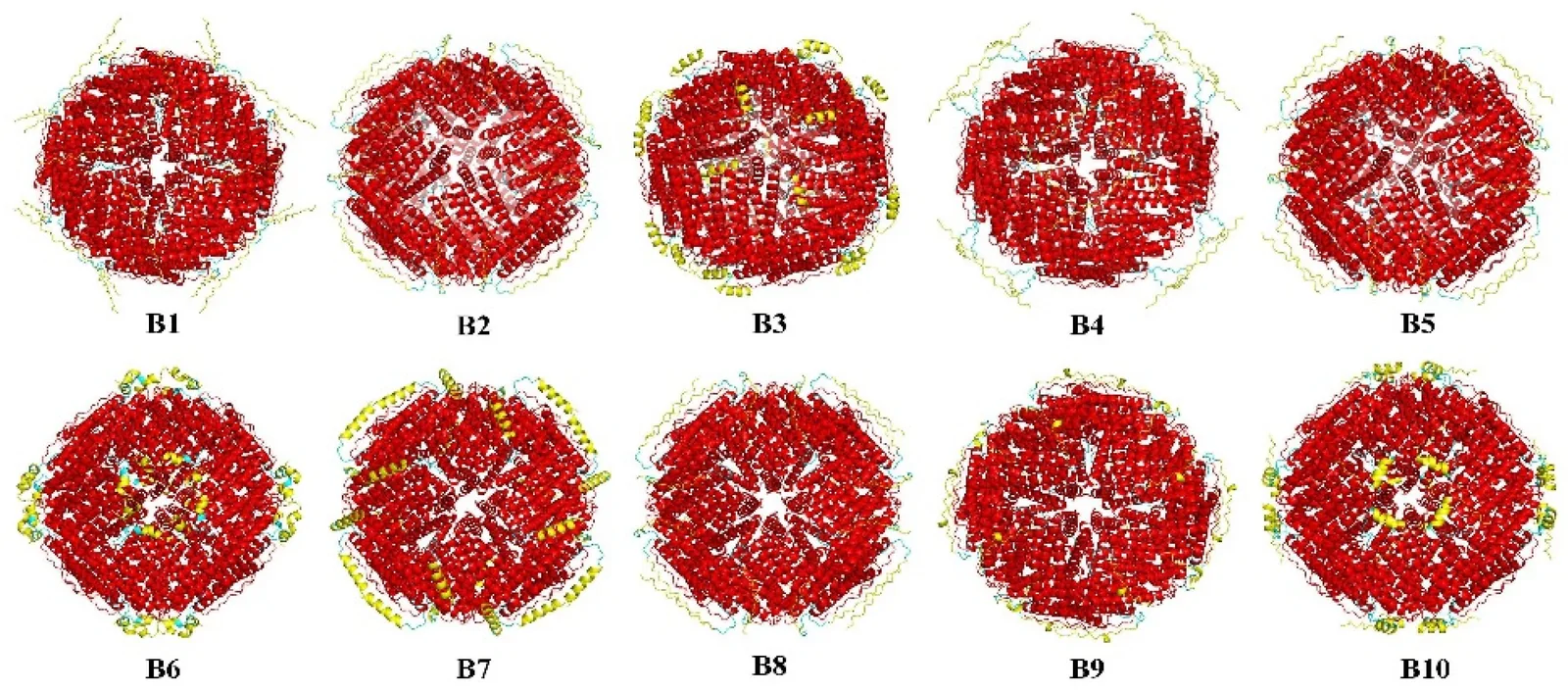
Three-dimensional structural models of B1–B10 ferritin nanocages predicted by AlphaFold 3 and visualized using PyMOL. The red sphere denotes the self-assembled 24-subunit ferritin core, with N-terminally fused linear B-cell epitopes (highlighted in yellow) fully exposed on the exterior surface. The linker sequence (GGGS)_2_ connecting each epitope to the ferritin core is shown in blue.

Initial in silico structural modeling using AlphaFold 3 and subsequent PyMOL visualization confirmed that the designed ferritin monomers assembled into typical nanocage-like structures, with the N-terminally fused linear B-cell epitopes displayed on the outer surface of the ferritin multimer (Figure 1). Based on this design, ten recombinant constructs, designated B1-ferritin through B10-ferritin, were generated by cloning the corresponding epitope-linker-ferritin sequences into the *pET-28a* vector. The complete aa sequences of all ten constructs are provided in Supplementary Table S2.

**Figure 2:**
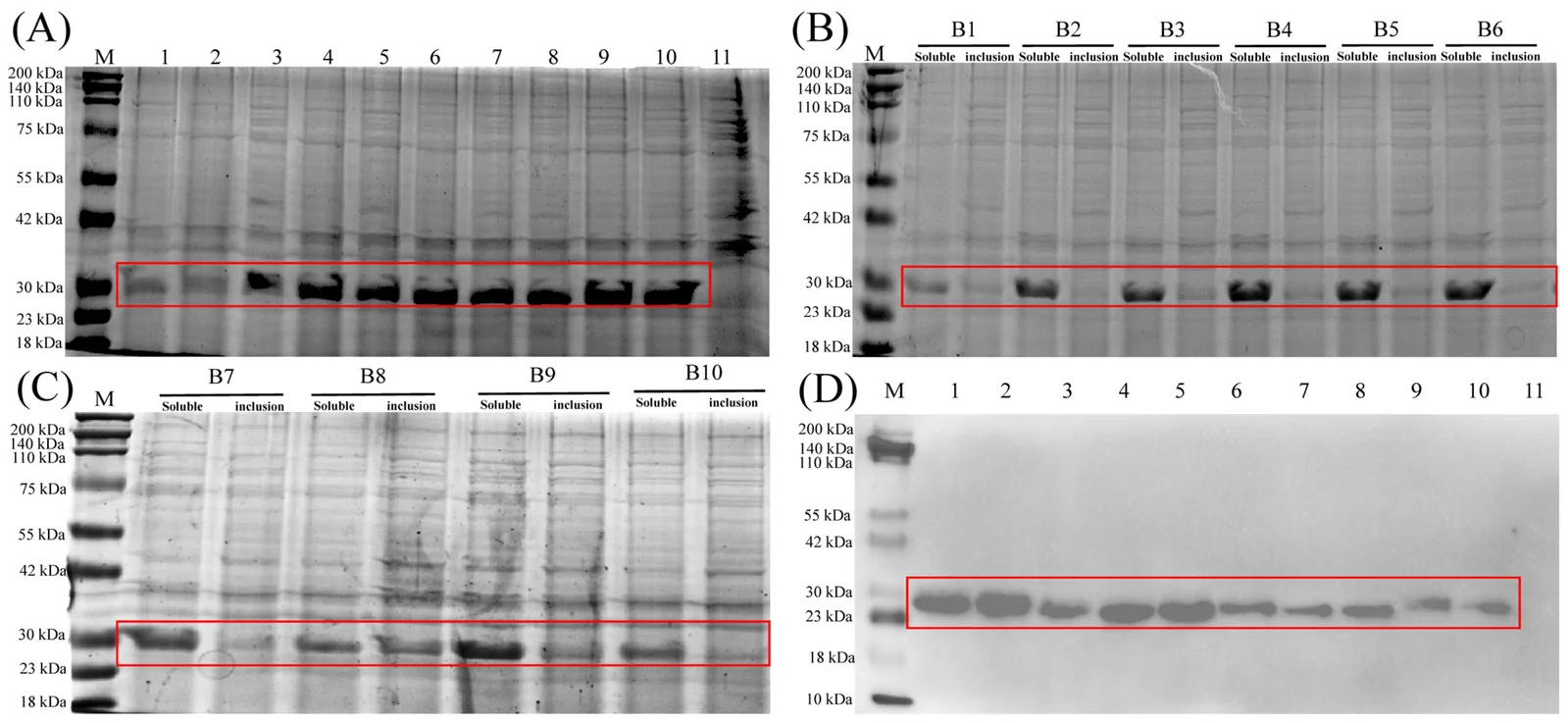
Structural design, expression (B1–B10). (**A**) SDS-PAGE analysis of the total expression profiles of B1–B10 constructs in *Escherichia coli* after induction optimization. Lane M: protein molecular weight marker; Lanes 1–10: induced total cell lysates of B1–B10 ferritin showing target bands at ~25 kDa (red box); Lane 11: uninduced negative control. (**B**,**C**) SDS-PAGE evaluation of the solubility of recombinant proteins, demonstrating robust soluble expression of B1–B10 constructs in the supernatant fraction (red box). (**D**) Western blot analysis of purified B1–B10 fusion proteins using anti-His antibody, verifying the antigenic integrity and correct molecular weight (~25 kDa, red box) of the ten nanoparticle display platforms.

Protein expression showed that induction with a low IPTG (0.1 mM) concentration at reduced temperature (16 °C) favored the soluble expression of the fusion proteins. SDS-PAGE analysis revealed prominent bands at the expected molecular weight of approximately 25 kDa (Figure 2A), and all ten B1–B10 ferritin proteins were detected mainly in the soluble fraction (Figure 2B,C). Western blot analysis of the purified proteins using an anti-His antibody yielded a single specific band at ~25 kDa for each construct (Figure 2D), confirming the successful expression and purification of the ten epitope-ferritin fusion proteins.

### 3.3. TEM Identification

**Figure 3:**
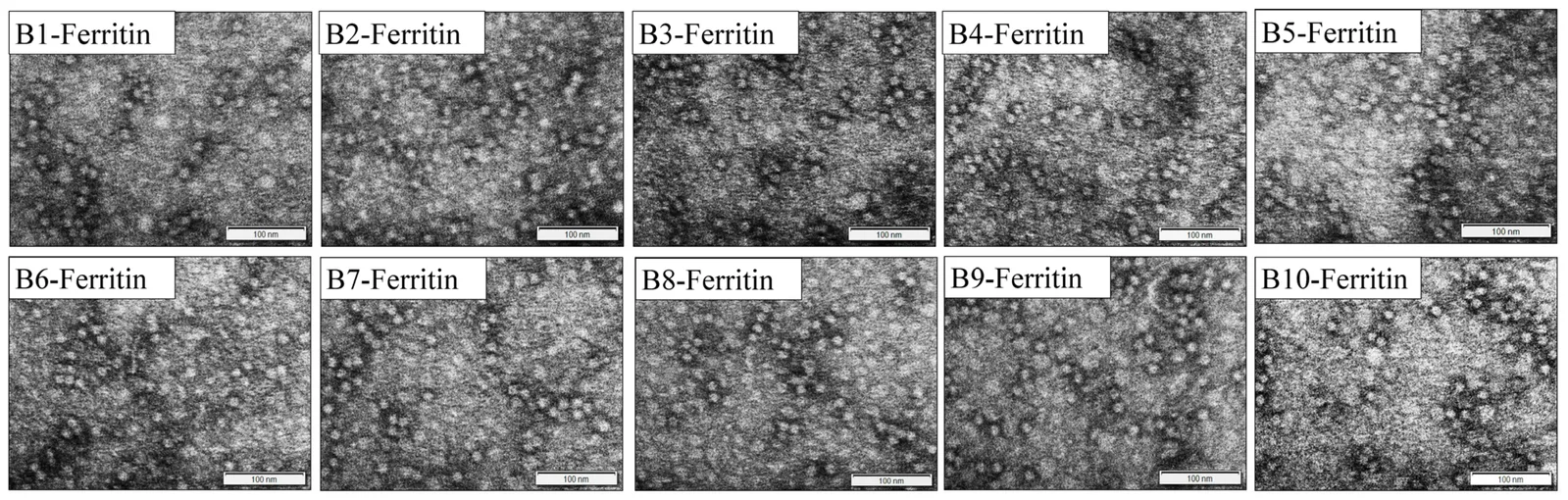
TEM images of purified B1–B10 epitope–ferritin nanoparticles. Samples (0.3 mg/mL) were negatively stained with 2% phosphotungstic acid and imaged by transmission electron microscopy (scale bar = 100 nm). All ten constructs formed uniform, spherical nanocages of consistent size, confirming successful self-assembly of the fusion proteins.

TEM analysis revealed that the B1–B10 ferritin fusion proteins spontaneously self-assembled into uniform, spherical nanostructures in vitro (Figure 3), confirming the intact self-assembly capability of the recombinant ferritin scaffolds. Particle size analysis using ImageJ showed that the average diameters of the B1–B10 ferritin nanoparticles were 12.07 ± 1.03, 13.72 ± 1.76, 12.40 ± 1.00, 13.12 ± 1.26, 12.54 ± 1.16, 12.38 ± 0.93, 12.21 ± 1.03, 12.73 ± 1.44, 11.79 ± 1.16, and 12.34 ± 0.98 nm, respectively, demonstrating a homogeneous nanoparticle size distribution.

### 3.4. Immunogenicity Evaluation of Fusion Proteins

**Figure 4:**
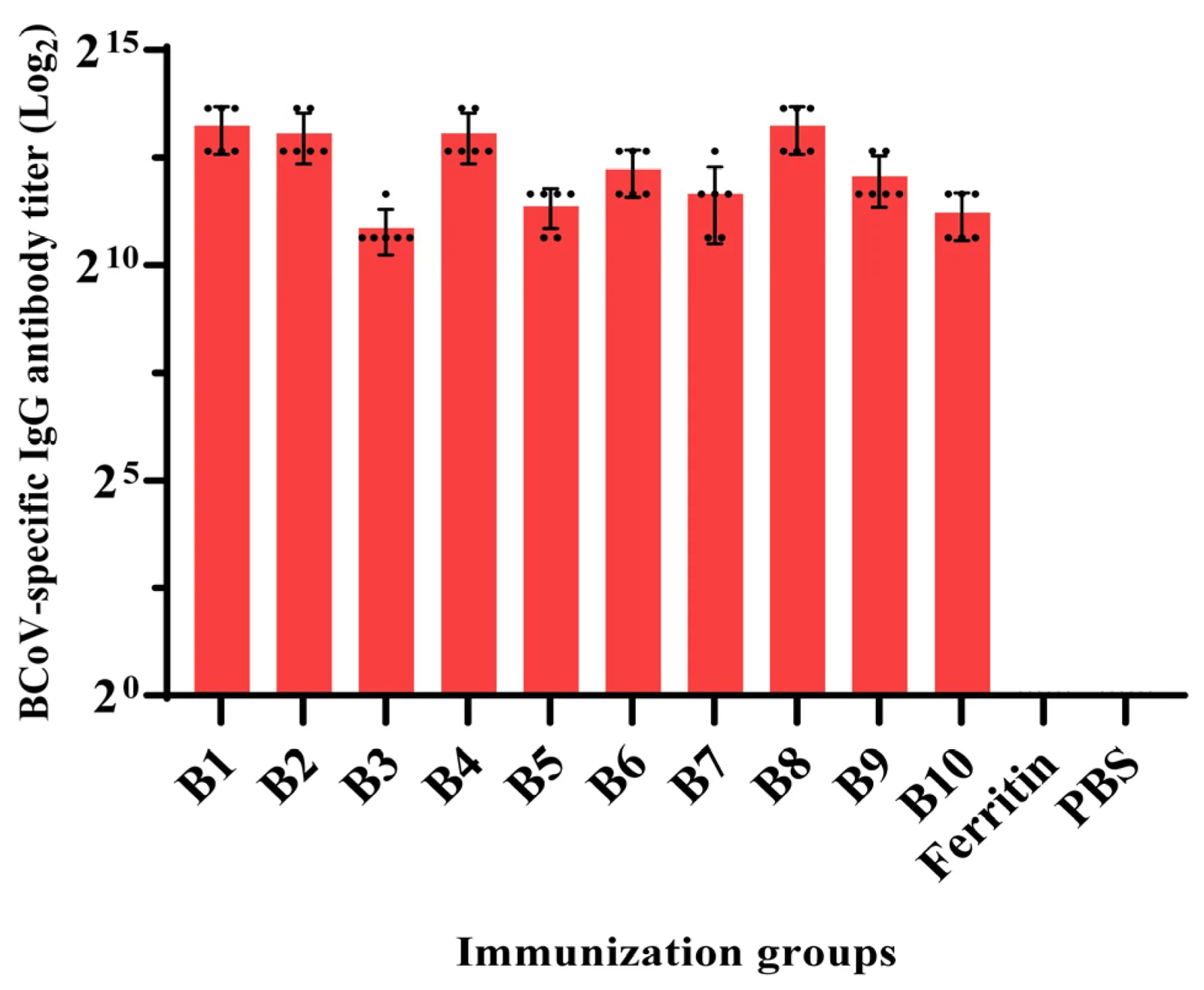
BCoV-specific serum IgG titers in mice immunized with B1–B10-ferritin nanoparticles, ferritin, or PBS were determined by indirect ELISA.

Indirect ELISA revealed that sera from mice immunized with all B1–B10 ferritin nanoparticles developed robust BCoV-specific IgG antibodies responses (Figure 4). Endpoint titers varied among the immunized groups, ranging from 1:2667 to 1:11,733, confirming that all ten constructs successfully elicited specific humoral immunity. In contrast, sera from the ferritin-only and PBS control groups showed no detectable BCoV-specific IgG. These results indicate that the elicited antibody responses were specifically driven by the displayed epitope sequences rather than the ferritin scaffold itself.

**Figure 5:**
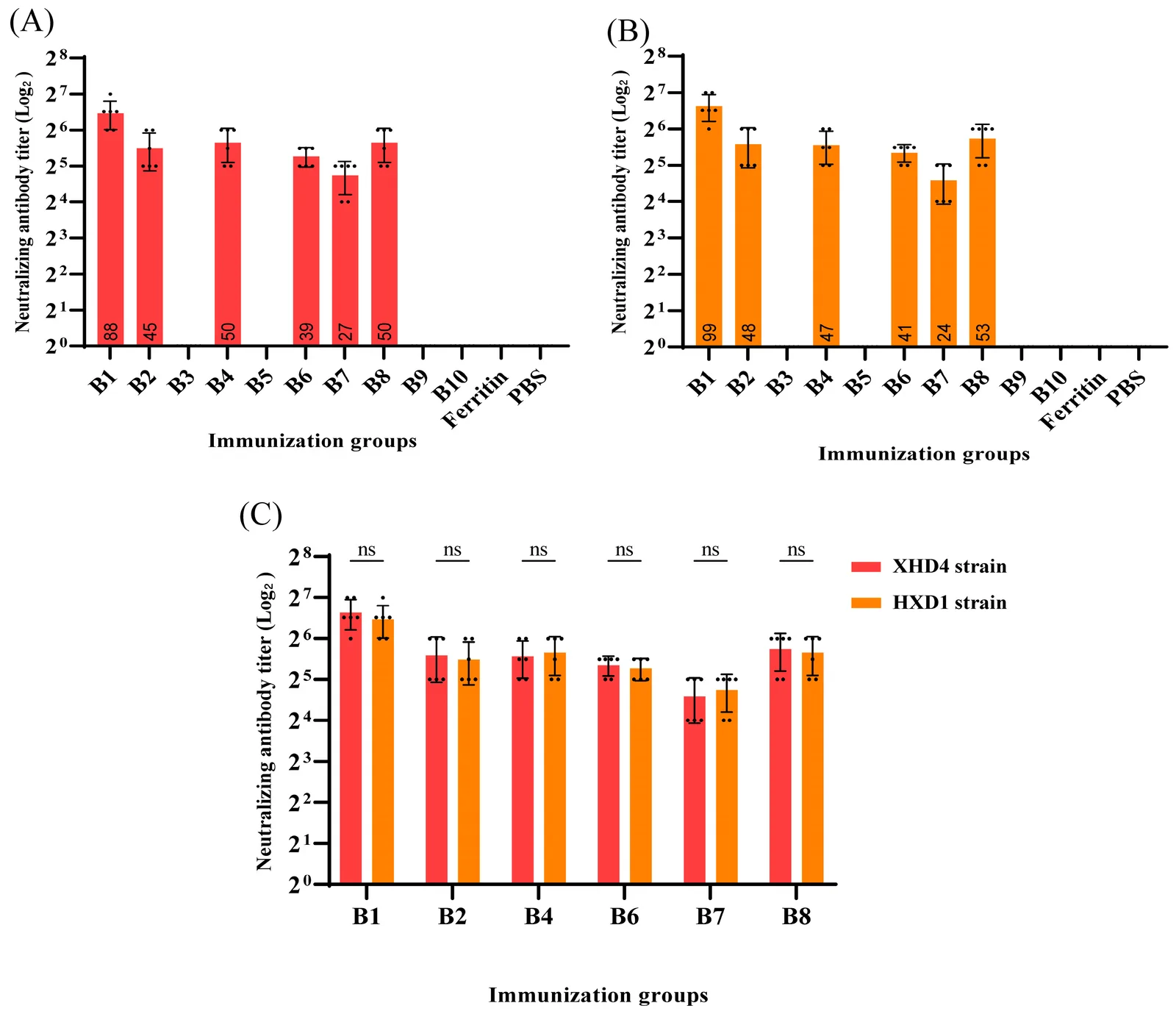
Evaluation of neutralizing antibody responses against enteric and respiratory strains induced by B1-B10-ferritin nanoparticles in mice. (**A**) Serum neutralizing antibody titers against the enteric BCoV strain (XHD4). The numbers at the base of the columns indicate the mean neutralizing antibody titers (reciprocal dilution) of the respective functional groups. (**B**) Serum neutralizing antibody titers against the respiratory BCoV strain (HXD1). The numbers at the base of the columns represent the mean neutralizing antibody titers of the respective functional groups. (**C**) Comparative analysis of neutralizing antibody capacities against the enteric (XHD4) and respiratory (HXD1) strains induced by the six functional epitope-displaying nanoparticles (B1, B2, B4, B6, B7, and B8). “ns” indicates no statistically significant difference (*p* > 0.05).

Serum neutralizing activity was further evaluated against two representative BCoV field strains, namely the enteric XHD4 strain and the respiratory HXD1 strain. No detectable neutralizing activity was observed in sera from mice immunized with B3-, B5-, B9-, or B10-ferritin nanoparticles, with titers remaining below the detection limit of 1:8. Similarly, the ferritin-only and PBS control groups showed no detectable neutralizing activity. In contrast, sera from mice immunized with B1-, B2-, B4-, B6-, B7-, and B8-ferritin nanoparticles successfully neutralized both BCoV strains to varying degrees (Figure 5A,B). For the XHD4 strain, the mean neutralizing antibody titers induced by B1, B2, B4, B6, B7, and B8 were 1:99, 1:48, 1:47, 1:41, 1:24, and 1:53, respectively. A similar neutralization trend was observed against the HXD1 strain, with mean titers of 1:88, 1:45, 1:50, 1:39, 1:27, and 1:50, respectively. Among these groups, B1-ferritin elicited the highest neutralizing titers against both strains, whereas B7-ferritin showed relatively lower yet consistent neutralizing activity. Comparative analysis showed no significant differences in neutralizing titers between the XHD4 and HXD1 strains for any of the six responsive epitope groups under the conditions tested (*p* > 0.05; Figure 5C). These results suggest that B1, B2, B4, B6, B7, and B8 represent promising conserved epitope candidates capable of inducing neutralizing antibody responses against both enteric and respiratory BCoV strains.

Of the six neutralizing epitopes candidates identified, five (B1, B2, B4, B6, and B8) were sequence-identical between XHD4 and HXD1, whereas the B7 epitope contained a single aa substitution in HXD1 relative to XHD4, the strain used for epitope prediction (Phe to Val; QEAIKFLNQSYINLKD in XHD4 versus QEAIKVLNQSYINLKD in HXD1). Despite this naturally occurring substitution, antisera against B7-ferritin neutralized both strains with comparable efficacy, and neutralizing titers did not differ significantly between the two strains for any of the six epitope groups (*p* > 0.05; Figure 5C). These findings corroborate the neutralizing activity against both clinical strains described above and indicate that these neutralizing epitopes are functionally conserved between the two clinical strains.

### 3.5. Conformational Recognition and 3D Structural Mapping of Neutralizing Epitopes on the BCoV S Protein

**Figure 6:**
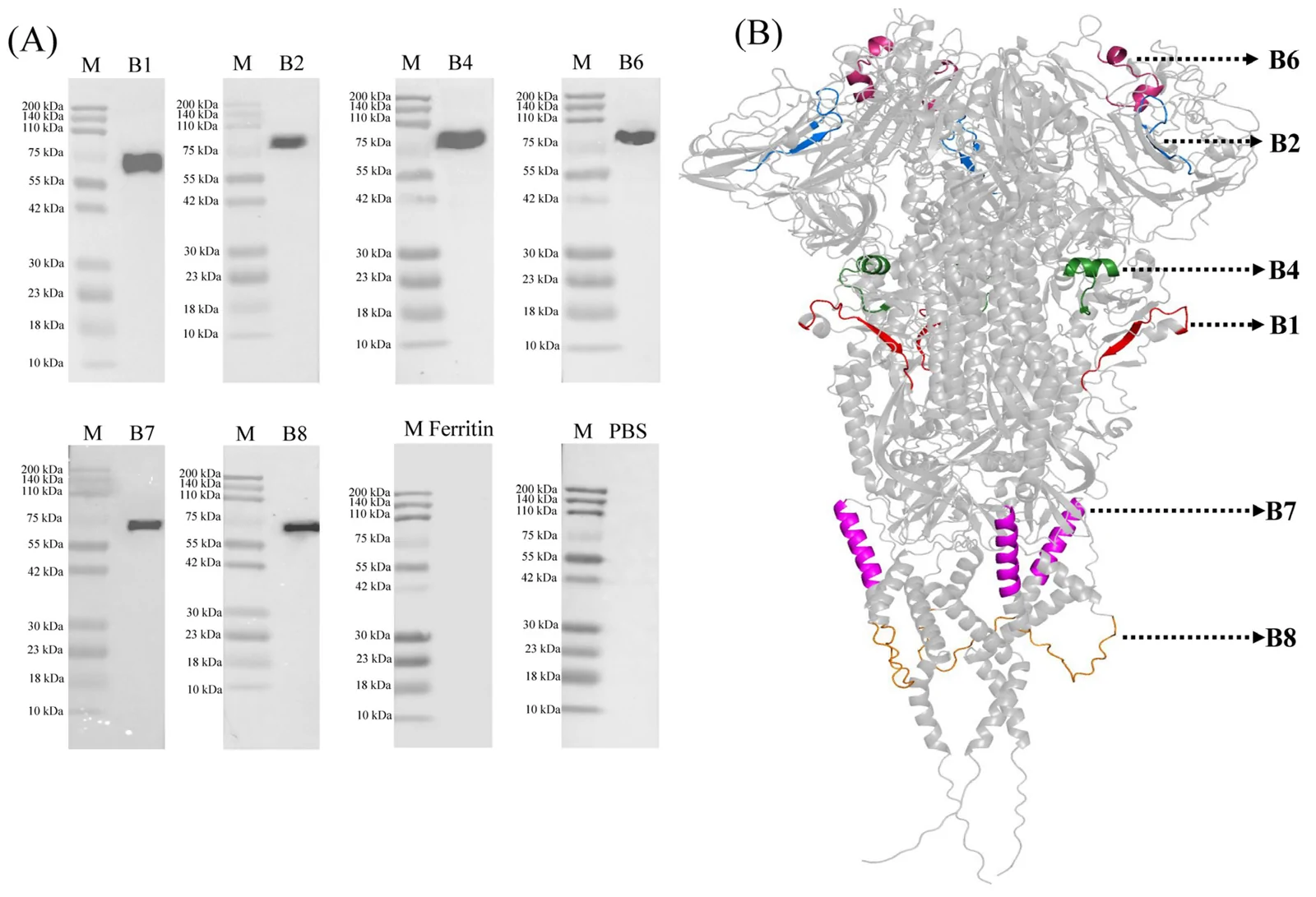
Validation of the localization of B-cell epitopes on the BCoV S protein. (**A**) Western blot analysis of ultracentrifugation-purified BCoV virions from infected cell culture supernatant, probed with antisera raised against each of the neutralizing epitopes (B1, B2, B4, B6, B7, and B8). Antisera raised against ferritin alone and against PBS served as negative controls. M, molecular weight marker. (**B**) Three-dimensional structure of the BCoV S trimer predicted by AlphaFold 3 and visualized in PyMOL, showing the epitope mapping of the six neutralizing epitope candidates (B1, B2, B4, B6, B7, and B8) on the S-trimer. The six epitopes that elicited neutralizing responses are color-coded as follows: B2 (NTD, blue), B4 (CTD, green), B6 (CTD, magenta), B1 (S1/S2 cleavage junction, spanning two residues in S1 and the remaining in S2, red), B8 (HR2, orange), and B7 (HR2, purple).

To confirm whether the neutralizing antibodies induced by the candidate epitopes could specifically recognize the native BCoV S protein, Western blot analysis was performed using purified intact virions harvested from infected cell culture supernatants (Figure 6A). The results demonstrated that antisera raised against all six candidate epitopes (B1, B2, B4, B6, B7, and B8) yielded specific single bands, whereas negative control antisera against ferritin alone or PBS showed no reactivity. Specifically, antisera against B2, B4, and B6 reacted with a protein band migrating at approximately 84 kDa, corresponding to the cleaved S1 subunit. In contrast, antisera against B1, B7, and B8 exclusively detected a band migrating at approximately 65 kDa, which aligns with the cleaved S2 subunit. The detection of B1 epitope at the ~65 kDa region is consistent with its sequence coverage, as it is predominantly located within the N-terminus of the S2 domain. These findings confirm that all six neutralizing epitope candidates are exposed on the native BCoV virion and successfully induce antibodies capable of recognizing the cleaved S1 and S2 subunits of the native S protein.

To elucidate the spatial distribution of these neutralizing epitopes, the six candidates were mapped onto the 3D homotrimeric structure of the BCoV S protein modeled by AlphaFold 3 (Figure 6B). The epitopes were distributed across critical functional domains of the S protein: B2 was localized to the N-terminal domain (NTD) of S1; B4 and B6 were mapped to the C-terminal domain (CTD) of S1; B1 was positioned at the S1/S2 cleavage junction, with two amino acids extending into the C-terminus of S1 and the remaining residues anchored within the N-terminus of S2; B7 and B8 were located within the heptad repeat 2 (HR2) region of the S2 subunit.

## 4. Discussion

BCoV is a major pathogen causing severe respiratory and enteric diseases in cattle, leading to substantial economic losses globally [1,2]. As the major surface antigen responsible for viral entry, the S glycoprotein is the principal target of host immune responses and conventional vaccine design [14,16]. However, antigenic variation in the S protein can facilitate escape from neutralizing antibodies and may reduce the effectiveness of conventional vaccines against emerging strains [3,4,5,6,7]. Therefore, vaccines targeting conserved B-cell epitopes associated with neutralizing activity represent a promising strategy for improving protection with potential cross-reactivity among BCoV strains. Similar approaches have been explored for other coronaviruses, including porcine epidemic diarrhea virus (PEDV) and HCoV-OC43 [25,26,27]. In this study, we combined in silico epitope prediction with a ferritin nanoparticle display platform and identified six conserved B-cell epitope candidates on the BCoV S protein that induced potent neutralizing antibody responses in mice. Importantly, these six neutralizing epitope candidates provide valuable leads for elucidating the functional roles of the BCoV S protein in viral entry and the mechanisms of antibody-mediated neutralization. Moreover, these validated conserved epitope candidates offer a solid foundation for the rational development of next-generation broad-spectrum vaccines, which are expected to retain efficacy across antigenically diverse BCoV lineages.

This study provides one of the first ferritin nanocage-based evaluations of conserved linear neutralizing epitope candidates in the BCoV S protein. A key challenge in epitope-based vaccine development is the limited immunogenicity and short in vivo persistence of isolated linear peptides [28]. To address this limitation, we employed *Helicobacter pylori* ferritin as a scaffold for epitope display. Ferritin spontaneously self-assembles into a highly ordered, 24-subunit spherical nanocage, providing an ideal platform for multivalent antigen presentation [20]. Consistent with the AlphaFold 3-based structural predictions and TEM analysis, the engineered B1–B10 epitope-ferritin proteins formed uniform nanoparticle structures with the fused epitopes positioned on the outer surface. Repetitive antigen display is known to resemble the ordered arrangement of epitopes on viral particles and can enhance B-cell activation by promoting B-cell receptor (BCR) clustering [29,30]. In line with this concept, the high IgG titers detected after two immunizations in mice suggest that ferritin-based multivalent display effectively enhanced the immunogenicity of these linear epitopes.

A notable finding of this study was the mismatch between binding antibody responses and neutralizing activity. Although all ten epitope-ferritin constructs induced high levels of BCoV-specific IgG, only six constructs B1, B2, B4, B6, B7, and B8, elicited potent neutralizing activity in vitro. One possible explanation is that the non-neutralizing, although conserved and antigenic, may not correspond to functionally vulnerable sites involved in receptor engagement, proteolytic activation, or membrane fusion, which aligns with the established concept that antibody binding does not necessarily predict virus neutralization [31]. In addition, their accessibility on the native prefusion spike trimer may be limited, or their local conformation on the native S protein may not be fully reproduced by linear peptide display on the ferritin scaffold, which is a known limitation of peptide-based epitope immunogen design [8,28]. Previous studies on coronavirus S proteins have shown that antigenic sites may differ substantially in surface exposure and neutralizing efficacy, with some epitopes being readily exposed whereas others are partially occluded [27,32]. Therefore, the absence of detectable neutralizing activity in the B3-, B5-, B9-, and B10-ferritin groups may reflect limited epitope accessibility, structural discrepancies, or a lack of direct involvement in critical steps of viral entry. Further studies using peptide competition assays, epitope-specific monoclonal antibodies, and mutational mapping will be needed to clarify the mechanisms underlying their non-neutralizing phenotype. It should be acknowledged that immunization with epitope–ferritin conjugates followed by polyclonal serum neutralization does not formally prove that neutralizing activity is driven exclusively by antibodies targeting the displayed epitope sequences. However, the ferritin-only control induced neither binding nor neutralizing antibodies, and only 6 of the 10 constructs elicited neutralizing responses despite comparable IgG titers across all groups. These observations argue against non-specific or scaffold-driven effects. Definitive confirmation of epitope-specific neutralization will require peptide competition or antibody adsorption assays in future studies.

In contrast to the non-neutralizing epitopes, mapping of the six neutralizing candidates onto the BCoV S protein showed that they were located within several functionally critical regions, which may elucidate their robust neutralizing activity. B2 (aa 122–137) is located within the NTD, the primary receptor-binding domain used by BCoV to engage host 9-O-acetylated sialic acid receptors [33]. Antibodies against this region may therefore interfere with viral attachment by sterically blocking receptor engagement. B4 (aa 291–306) and B6 (aa 445–460) map to the CTD. Although the NTD is considered the main receptor-binding region of BCoV, studies on related coronaviruses suggest that antibodies targeting the CTD may limit the conformational flexibility of the S protein and thereby interfere with downstream steps of viral entry [34].

Neutralization by antibodies targeting the S2 subunit is generally associated with interference in proteolytic activation or membrane fusion. B1 (aa 767–782) spans the critical S1/S2 cleavage site, a region required for protease-mediated activation of the S protein. Antibodies recognizing this epitope may reduce protease accessibility to the cleavage site and thereby impair the transition of S into a fusion-competent state [35]. B7 (aa 1281–1297) and B8 (aa 1256–1272) are located within the highly conserved HR2 region. During viral entry, HR2 interacts with HR1 to form the six-helix bundle (6-HB) that drives fusion between the viral and cellular membranes. Antibodies binding to HR2 could potentially disrupt this refolding process via steric hindrance, as has been reported for several coronaviruses [32]. However, these proposed mechanisms require further validation using monoclonal antibodies, peptide-blocking assays, or mutational analyses. Together, the structural mapping of these six neutralizing epitope candidates provides valuable insights for future structure-guided BCoV vaccine design.

Furthermore, the reactivity of the neutralizing antisera was validated against purified BCoV virions via Western blotting. The specific detection of single bands corresponding to the cleaved S1 subunit (~84 kDa) for B2, B4, and B6-ferritin, and the S2 subunit (~65 kDa) for B1, B7, and B8-ferritin confirms that these antibodies specifically recognize the native components of the virion S protein.

Despite these encouraging findings, several limitations should be addressed in future studies. First, although the epitope candidates were selected using in silico linear epitope prediction and their reactivity with native S protein subunits was confirmed via Western blotting, their precise high-resolution structural details when displayed on the nanocage remain to be elucidated. Future structural studies, such as cryo-electron microscopy (cryo-EM) analysis of epitope-ferritin complexes or peptide competition ELISA, will be valuable to further define the exact conformational states and binding affinities of these epitopes. Second, the neutralizing evaluation in this study was conducted using two representative clinical isolates (XHD4 and HXD1). Although these strains represent the principal enteric and respiratory forms of BCoV infection and belong to genotype GIIb (the predominant lineage in China), they do not represent the full genetic diversity of BCoV isolates globally. Testing additional phylogenetic lineages and field variants in future work will help establish the breadth of neutralization and validate cross-lineage protective potential. Third, sample size (n = 6 per group) may have limited the statistical power to detect subtle quantitative differences between groups. Nevertheless, post hoc power analysis confirmed that the large biological effect sizes observed (Cohen’s d > 2.0) provided a statistical power (1 − β) exceeding 0.85 for both indirect ELISA and neutralization assays. This group size was sufficient to establish the principal findings of this study, which rely on large and consistent effects rather than subtle differences: the induction of binding antibodies across all constructs, the restriction of neutralizing activity to six of the ten epitopes, and the marked differences between responder and non-responder groups. These qualitative, large-effect outcomes were consistently detected by the indirect ELISA and the live-virus neutralization assays used here, indicating that the sample size was adequate for the tests on which the main conclusions rest. Future preclinical evaluations in the target bovine host with larger cohorts will be valuable to further substantiate protective efficacy. Fourth, given that BCoV infects both the respiratory and intestinal tracts, future studies should also examine mucosal immunization strategies, such as intranasal delivery, to induce local secretory IgA responses. This is particularly relevant because subcutaneous immunization with Montanide ISA 201 mainly induces systemic IgG responses. In summary, the identification of these six conserved neutralizing epitope candidates, together with the robust antigen-display capacity of the self-assembling ferritin platform, provides a solid basis for the rational design of next-generation vaccines against diverse BCoV isolates and possibly related coronaviruses.

## 5. Conclusions

In conclusion, our study identified six highly conserved B-cell epitope candidates in the BCoV S protein that were capable of inducing neutralizing antibody responses against both representative clinical BCoV strains. The ferritin nanoparticle display platform supported multivalent presentation of these epitopes and elicited robust BCoV-specific antibody responses in mice. These findings provide valuable insights into the conserved antigenic regions of the BCoV S protein and offer a rational framework for the development of epitope-focused BCoV vaccine candidates. Further validation in the natural host and against a broader panel of BCoV isolates will be required to assess their protective efficacy.

## Figures and Tables

**Table 1:** Prediction and Screening of Linear B-cell Epitopes on BCoV S Protein.

Name	Linear B-Cell Epitope Sequence	Start Position	ABCPred Score	Antigenicity	Toxicity	Allergenicity	Conservation
B1	RRSITTGYRFTNFEPF	767	0.92	1.282	-	-	98.37%
B2	FPAITIGSTFVNTSYS	122	0.84	1.045	-	-	99.80%
B3	SPLNWERKTFSNCNFN	344	0.83	1.259	-	-	99.93%
B4	MSEIKCKTLSIAPSTG	291	0.79	1.437	-	-	99.84%
B5	AVIGDLKCTTVSINDV	14	0.78	1.491	-	-	99.46%
B6	PSTWNRRFGFTEQSVF	445	0.73	1.108	-	-	98.85%
B7	QEAIKFLNQSYINLKD	1281	0.71	1.197	-	-	99.59%
B8	SVAPDLSLDYINVTFL	1256	0.71	1.343	-	-	98.65%
B9	LKDGVNFNVDDINFSP	883	0.68	1.363	-	-	99.06%
B10	NAVDCKSDFMSEIKCK	282	0.68	1.035	-	-	99.71%

Note: “-” indicates negative.

## Data Availability

The data used to support the findings of this study are included within the article.

## Supplementary Materials

The following supporting information can be downloaded at https://www.mdpi.com/article/10.3390/ani16162598/s1, Figure S1: Phylogenetic analysis of the *S* gene sequences of BCoV strains. Maximum-likelihood phylogenies of all BCoV *S* gene sequences retrieved from GenBank were inferred under the best-fit substitution model TIM+F+I+R4, with 1,000 bootstrap replicates, and resolved into the GIa, GIb, GIIa, and GIIb genotypes. The red circle (●) marks the enteric strain XHD4 (GenBank OR621178) sequenced in this study; the green circle (●) marks the respiratory strain HXD1 (GenBank OR12022); and black circles (●) denote BCoV reference sequences from China; Table S1: Predicted linear B-cell epitopes in the BCoV Spike protein; Table S2: Amino acid sequences of the ten B1~B10 epitope-ferritin fusion constructs.

## References

[1] Bok M., Miño S., Rodriguez D., Badaracco A., Nuñes I., Souza S.P., Bilbao G., Uriarte E.L., Galarza R., Vega C. (2015). Molecular and antigenic characterization of bovine Coronavirus circulating in Argentinean cattle during 1994–2010. Vet. Microbiol..

[2] Suzuki T., Otake Y., Uchimoto S., Hasebe A., Goto Y. (2020). Genomic Characterization and Phylogenetic Classification of Bovine Coronaviruses Through Whole Genome Sequence Analysis. Viruses.

[3] Franzo G., Drigo M., Legnardi M., Grassi L., Pasotto D., Menandro M.L., Cecchinato M., Tucciarone C.M. (2020). Bovine Coronavirus: Variability, Evolution, and Dispersal Patterns of a No Longer Neglected Betacoronavirus. Viruses.

[4] Kanno T., Ishihara R., Hatama S., Uchida I. (2013). Antigenic variation among recent Japanese isolates of bovine coronaviruses belonging to phylogenetically distinct genetic groups. Arch. Virol..

[5] Saif L.J. (2010). Bovine Respiratory Coronavirus. Vet. Clin. N. Am. Food Anim. Pract..

[6] Đurić M. (2022). Bovine Coronavirus Infection.

[7] Fulton R., d’Offay J., Landis C., Miles D., Smith R., Saliki J., Ridpath J., Confer A., Neill J., Eberle R. (2016). Detection and characterization of viruses as field and vaccine strains in feedlot cattle with bovine respiratory disease. Vaccine.

[8] Biavasco R., De Giovanni M. (2022). The relative positioning of B and T cell epitopes drives immunodominance. Vaccine.

[9] De Groot A.S., Moise L., Terry F., Gutierrez A.H., Hindocha P., Richard G., Hoft D.F., Ross T.M., Noe A.R., Takahashi Y. (2020). Better epitope discovery, precision immune engineering, and accelerated vaccine design using immunoinformatics tools. Front. Immunol..

[10] Sette A., Crotty S. (2021). Adaptive immunity to SARS-CoV-2 and COVID-19. Cell.

[11] Wang C., Hesketh E.L., Shamorkina T.M., Li W., Franken P.J., Drabek D., van Haperen R., Townend S., van Kuppeveld F.J.M., Grosveld F. (2022). Antigenic structure of the human coronavirus OC43 spike reveals exposed and occluded neutralizing epitopes. Nat. Commun..

[12] Wang C.Y., Peng W.-J., Kuo B.-S., Ho Y.-H., Wang M.-S., Yang Y.-T., Chang P.-Y., Shen Y.-H., Hwang K.-P. (2023). Toward a pan-SARS-CoV-2 vaccine targeting conserved epitopes on spike and non-spike proteins for potent, broad and durable immune responses. PLoS Pathog..

[13] Kin N., Miszczak F., Diancourt L., Caro V., Moutou F., Vabret A., Gouilh M.A. (2016). Comparative molecular epidemiology of two closely related coronaviruses, bovine coronavirus (BCoV) and human coronavirus OC43 (HCoV-OC43), reveals a different evolutionary pattern. Infect. Genet. Evol..

[14] Vlasova A.N., Saif L.J. (2021). Bovine coronavirus and the associated diseases. Front. Vet. Sci..

[15] Duraisamy N., Khan M.Y., Shah A.U., Elalaoui R.N., Cherkaoui M., Hemida M.G. (2024). Machine learning tools used for mapping some immunogenic epitopes within the major structural proteins of the bovine coronavirus (BCoV) and for the in silico design of the multiepitope-based vaccines. Front. Vet. Sci..

[16] Jiang Q., Ma Z., Min F., Ding X., Liang Y., Wang J., Liu L., Li N., Sun Y., Zhong Q. (2024). Screening of bovine coronavirus multiepitope vaccine candidates: An immunoinformatics approach. Transbound. Emerg. Dis..

[17] Yoo D., Deregt D. (2001). A single amino acid change within antigenic domain II of the spike protein of bovine coronavirus confers resistance to virus neutralization. Clin. Diagn. Lab. Immunol..

[18] Saha S., Raghava G.P.S. (2006). Prediction of continuous B-cell epitopes in an antigen using recurrent neural network. Proteins Struct. Funct. Bioinform..

[19] Li W., Sun T., Li M., He Y., Li L., Wang L., Wang H., Li J., Wen H., Liu Y. (2022). GNIFdb: A neoantigen intrinsic feature database for glioma. Database.

[20] Kanekiyo M., Wei C.-J., Yassine H.M., McTamney P.M., Boyington J.C., Whittle J.R.R., Rao S.S., Kong W.-P., Wang L., Nabel G.J. (2013). Self-assembling influenza nanoparticle vaccines elicit broadly neutralizing H1N1 antibodies. Nature.

[21] Pattnaik A., Sahoo B.R., Struble L.R., Borgstahl G.E.O., Zhou Y., Franco R., Barletta R.G., Osorio F.A., Petro T.M., Pattnaik A.K. (2023). A ferritin nanoparticle-based Zika virus vaccine candidate induces robust humoral and cellular immune responses and protects mice from lethal virus challenge. Vaccines.

[22] Vu M.N., Pilkington E.H., Lee W.S., Tan H.X., Davis T.P., Truong N.P., Kent S.J., Wheatley A.K. (2023). Engineered ferritin nanoparticle vaccines enable rapid screening of antibody functionalization to boost immune responses. Adv. Healthc. Mater..

[23] Langel S.N., Paim F.C., Alhamo M.A., Buckley A., Van Geelen A., Lager K.M., Vlasova A.N., Saif L.J. (2019). Stage of gestation at porcine epidemic diarrhea virus infection of pregnant swine impacts maternal immunity and lactogenic immune protection of neonatal suckling piglets. Front. Immunol..

[24] Shah A.U., Gauger P., Hemida M.G. (2025). Isolation and molecular characterization of an enteric isolate of the genotype-Ia bovine coronavirus with notable mutations in the receptor binding domain of the spike glycoprotein. Virology.

[25] Okda F.A., Lawson S., Singrey A., Nelson J., Hain K.S., Joshi L.R., Christopher-Hennings J., Nelson E.A., Diel D.G. (2017). The S2 glycoprotein subunit of porcine epidemic diarrhea virus contains immunodominant neutralizing epitopes. Virology.

[26] Pinto D., Sauer M.M., Czudnochowski N., Low J.S., Tortorici M.A., Housley M.P., Noack J., Walls A.C., Bowen J.E., Guarino B. (2021). Broad betacoronavirus neutralization by a stem helix–specific human antibody. Science.

[27] Sauer M.M., Tortorici M.A., Park Y.-J., Walls A.C., Homad L., Acton O.J., Bowen J.E., Wang C., Xiong X., de van der Schueren W. (2021). Structural basis for broad coronavirus neutralization. Nat. Struct. Mol. Biol..

[28] Malonis R.J., Lai J.R., Vergnolle O. (2019). Peptide-based vaccines: Current progress and future challenges. Chem. Rev..

[29] Mohsen M.O., Zha L., Cabral-Miranda G., Bachmann M.F. (2017). Major findings and recent advances in virus–like particle (VLP)-based vaccines. Semin. Immunol..

[30] Kelly H.G., Kent S.J., Wheatley A.K. (2019). Immunological basis for enhanced immunity of nanoparticle vaccines. Expert Rev. Vaccines.

[31] Klasse P.J. (2014). Neutralization of virus infectivity by antibodies: Old problems in new perspectives. Adv. Biol..

[32] Wang C., van Haperen R., Gutiérrez-Álvarez J., Li W., Okba N.M.A., Albulescu I., Widjaja I., van Dieren B., Fernandez-Delgado R., Sola I. (2021). A conserved immunogenic and vulnerable site on the coronavirus spike protein delineated by cross-reactive monoclonal antibodies. Nat. Commun..

[33] Peng G., Xu L., Lin Y.-L., Chen L., Pasquarella J.R., Holmes K.V., Li F. (2012). Crystal structure of bovine coronavirus spike protein lectin domain. J. Biol. Chem..

[34] Hulswit R.J.G., de Haan C.A.M., Bosch B.J. (2016). Coronavirus spike protein and tropism changes. Adv. Virus Res..

[35] Millet J.K., Whittaker G.R. (2015). Host cell proteases: Critical determinants of coronavirus tropism and pathogenesis. Virus Res..

