# Supplementary material for: Identification of Conserved B-Cell Epitope Candidates Associated with Neutralizing Activity of Bovine Coronavirus Spike Protein
Source: Animals (Basel). 2026 Aug 20;16(16):2598. doi: 10.3390/ani16162598 (PMC13508896; doi:10.3390/ani16162598)
Supplement: Supplementary file 1 [file animals-16-02598-s001.zip › Supplementary Figure S1.pdf]

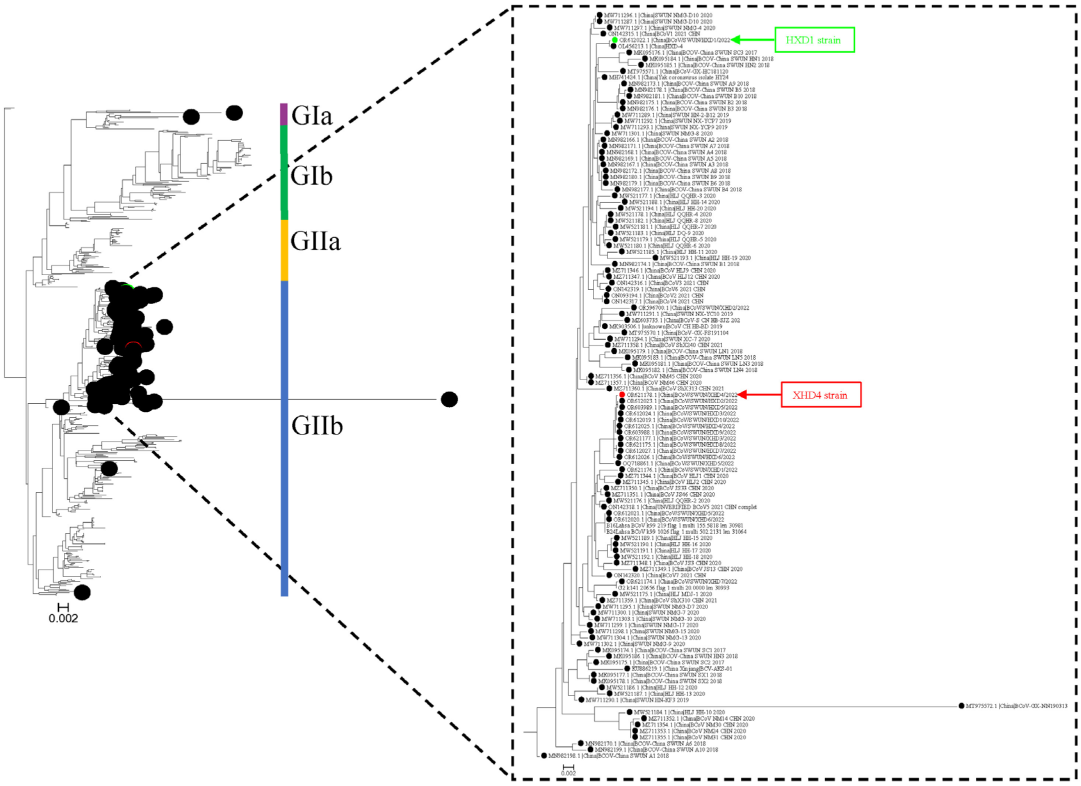

Supplementary Figure S1. Phylogenetic analysis of the S gene sequences of BCoV strains. Maximum-likelihood phylogenies of all BCoV S gene sequences retrieved from GenBank were inferred under the best-fit substitution model TIM+F+I+R4, with 1,000 bootstrap replicates, and resolved into the GIa, GIb, GIIa, and GIIb genotypes. The red circle (●) marks the enteric strain XHD4 (GenBank OR621178) sequenced in this study; the green circle (●) marks the respiratory strain HXD1 (GenBank OR12022); and black circles (●) denote BCoV reference sequences from China.
